# Searching for Best Predictors of Paralinguistic Comprehension and Production of Emotions in Communication in Adults With Moderate Intellectual Disability

**DOI:** 10.3389/fpsyg.2022.884242

**Published:** 2022-07-08

**Authors:** Gordana Calić, Nenad Glumbić, Mirjana Petrović-Lazić, Mirjana Đorđević, Tatjana Mentus

**Affiliations:** Faculty of Special Education and Rehabilitation, University of Belgrade, Belgrade, Serbia

**Keywords:** intellectual ability, moderate intellectual disability, paralinguistic comprehension, paralinguistic production, receptive language ability, acoustic features of voice, Serbian language

## Abstract

Paralinguistic comprehension and production of emotions in communication include the skills of recognizing and interpreting emotional states with the help of facial expressions, prosody and intonation. In the relevant scientific literature, the skills of paralinguistic comprehension and production of emotions in communication are related primarily to receptive language abilities, although some authors found also their correlations with intellectual abilities and acoustic features of the voice. Therefore, the aim of this study was to investigate which of the mentioned variables (receptive language ability, acoustic features of voice, intellectual ability, social-demographic), presents the most relevant predictor of paralinguistic comprehension and paralinguistic production of emotions in communication in adults with moderate intellectual disabilities (MID). The sample included 41 adults with MID, 20–49 years of age (*M* = 34.34, SD = 7.809), 29 of whom had MID of unknown etiology, while 12 had Down syndrome. All participants are native speakers of Serbian. Two subscales from The Assessment Battery for Communication – Paralinguistic comprehension of emotions in communication and Paralinguistic production of emotions in communication, were used to assess the examinees from the aspect of paralinguistic comprehension and production skills. For the graduation of examinees from the aspect of assumed predictor variables, the following instruments were used: Peabody Picture Vocabulary Test was used to assess receptive language abilities, Computerized Speech Lab (“Kay Elemetrics” Corp., model 4300) was used to assess acoustic features of voice, and Raven’s Progressive Matrices were used to assess intellectual ability. Hierarchical regression analysis was applied to investigate to which extent the proposed variables present an actual predictor variables for paralinguistic comprehension and production of emotions in communication as dependent variables. The results of this analysis showed that only receptive language skills had statistically significant predictive value for paralinguistic comprehension of emotions (β = 0.468, *t* = 2.236, *p* < 0.05), while the factor related to voice frequency and interruptions, form the domain of acoustic voice characteristics, displays predictive value for paralinguistic production of emotions (β = 0.280, *t* = 2.076, *p* < 0.05). Consequently, this study, in the adult population with MID, evidenced a greater importance of voice and language in relation to intellectual abilities in understanding and producing emotions.

## Introduction

According to DSM-5 (American Psychiatric Association, 2013), intellectual disability (ID) is defined as a disability characterized by significant limitations both in intellectual functioning and adaptive behavior, manifested in conceptual, social, and practical adaptive skills. The social aspect of adaptive skills, among other things, includes empathy, social-emotional reasoning, establishing interpersonal interactions, etc. (American Psychiatric Association, 2013). The ability to recognize and produce emotions contributes to successful social adaptation and facilitates coping in a social environment (Kashani-Vahid et al., 2018). On the other hand, difficulties in understanding emotions may affect social adaptation and integration of people with ID (Trentacosta and Fine, 2010) and cause aggressive outbursts (Matheson and Jahoda, 2005). People with ID usually have difficulties in recognizing emotions, which persists in adulthood (Scotland et al., 2015, 2016). The persisting problems in understanding emotions also affect finding and keeping a job, self-esteem, and thus the overall quality of life of these people (Banks et al., 2010). Since there are various adverse effects of reduced emotion recognition abilities in people with ID, research studies aimed at detecting the predictors of these abilities may be useful, as they may result in guidelines for targeted and effective interventions. Nowadays, this kind of research is increasingly gaining importance as a support for teaching models for automatic emotion recognition, i.e., the creation of technological assistive devices that facilitate the treatment of children and adults with neurodevelopmental disorders (Ma et al., 2019; Martinez-Martin et al., 2020; Landowska et al., 2022).

Paralinguistic elements include various sounds, tones, crying, laughing, sobbing, speech speed and pitch, rhythm and intonation (Rot, 2004), as well as facial expressions (e.g., in the eyes and lips region – eyes widening) (Angeleri et al., 2012; Tomić, 2014). They also answer the question: “How has something been said?”. Therefore, paralinguistic comprehension of emotions could be viewed as the skill to precisely recognize and interpret an emotional state based on non-verbal clues, such as facial expressions, prosody, and/or body language (Angeleri et al., 2012, 2016). On the other hand, paralinguistic production of emotions involves expressing emotions by using non-linguistic aspects of speech, such as mime and prosody (Angeleri et al., 2012, 2016). Many studies show that people with ID perform worse on tasks involving paralinguistic recognition, comprehension, and production of emotions, than typically developing people (Hippolyte et al., 2008; Scotland et al., 2015, 2016; Murray et al., 2019; McKenzie et al., 2021). There are several hypotheses that can potentially explain the mentioned difficulties (e.g., Albanese et al., 2010; Beck et al., 2012; Mazzoni et al., 2020). However, it is still not completely clear what these skills are most closely related to both in typically developing people and people with ID.

A deficit in language skills, especially receptive skills, in people with ID may be related to difficulties in understanding emotions. Emotional recognition includes knowledge of emotions acquired from life experience, just as receptive vocabulary includes knowledge of concepts acquired from experience (Beck et al., 2012). Joyce et al. (2006) show that adults with ID are better at recognizing than producing emotions, and that these abilities are related to receptive language skills. Also, the results of some studies indicate a significant positive correlation between receptive language skills and recognizing certain emotions from facial expressions in adults with Down syndrome (DS^1^), while such correlation was not found in typically developing adults (Hippolyte et al., 2008).

Compared to language skills, the literature on prosodic features of voice in people with ID is not so extensive. Still, people with ID express certain changes in acoustic voice characteristics compared to typically developing people, both in childhood and in adulthood (Lee et al., 2009; Amadó et al., 2016; Becker et al., 2017; O’ Leary et al., 2019). Various research studies on typically developing people show that acoustic voice characteristics [e.g., Pitch, Fundamental frequency (F0), F0 contour, Jitter, Intensity, Attack, Pauses, etc.] are significant factors in recognizing emotions (Scherer et al., 1991; Banse and Scherer, 1996; Juslin and Laukka, 2003). Numerous studies have examined how a listener recognizes emotions from acoustic voice characteristics in situations where trained actors express emotional states while reading syllables or meaningless texts (e.g., Banse and Scherer, 1996). Basic emotions are best recognized (happiness, sadness, anger, surprise, fear, and disgust) when the actors who express emotions belong to the same culture as the listeners (Juslin and Laukka, 2003; Pell et al., 2009). In addition, research shows that vocal sighs (expressed through laughter, a scream, an exclamation, etc.) contribute to recognizing different emotions in given speech situations (Sauter and Scott, 2007; Simon-Thomas et al., 2009; Sauter et al., 2010; Laukka et al., 2013). Based on the information from studies on typical population, the question arises as to whether and to what extent these acoustic characteristics affect paralinguistic recognition and production in people with ID.

Limitations in overall intellectual functioning may also contribute to difficulties in understanding and producing emotions in people with ID, as indicated by some authors in this field (Moore, 2001). Also, IQ is negatively correlated with processing emotional information in people with borderline intellectual functioning (Smirni et al., 2019). One explanation is that emotional and cognitive intelligence share a neuro-functional-anatomical network and that explains these correlations (Barbey et al., 2014, according to Smirni et al., 2019). Higher non-verbal IQ was also associated with better emotional understanding in the population of participants with autism with different levels of cognitive functioning (Salomone et al., 2019). The relationship between non-verbal intelligence and understanding of emotions certainly exists, but it is not simple and age mediates it (Albanese et al., 2010). Matheson and Jahoda (2005) show that emotion recognition is associated with receptive language skills but not IQ. However, some authors (Beck et al., 2012) suggest that intellectual abilities could also account for the relation between receptive vocabulary and recognizing emotions. The same authors propose further examination of the role of intelligence in the development of emotional competencies.

Age is another significant variable in recognizing emotions. It has been shown that older typically developing adults perform worse on emotion recognition tasks than younger adults (Sullivan et al., 2015). In a meta-analysis that examined the relationship between intelligence and emotional recognition in adults of a typical population, it was found that the relationship exists, but that age also plays a significant role in it. One explanation is that in the elderly, the stronger connection between intelligence and emotion recognition can be explained by the hypothesis of cognitive dedifferentiation, which claims that the structure of individuals’ cognitive abilities becomes less differentiated in old age (Schlegel et al., 2020). Furthermore, some research studies show that older adults produce emotions more slowly than younger adults (Dupuis and Pichora-Fuller, 2010). Apart from age, gender differences have also been found. Women are generally more successful in recognizing emotions than men (Sullivan et al., 2015; Gonçalves et al., 2018). Similarly, the ability to recognize emotions decreases with age in adults with ID (Matheson and Jahoda, 2005). An older study (McKenzie et al., 2000) on a sample of adults with mild and moderate ID showed that younger participants and those with milder ID were better at recognizing emotions.

The literature shows that so far researchers have focused more on examining people with autism spectrum disorder than people with ID with regard to paralinguistic comprehension and production. Compared to language and cognitive abilities, the field of social-emotional skills in this population has been less frequently examined (Pochon et al., 2017). Research has mainly examined emotion recognition abilities based on facial expressions and prosodic elements of speech in people with ID compared to typically developing population, mostly at a younger age (Fernández-Alcaraz et al., 2010; Cebula et al., 2017; Pochon et al., 2017; Martínez-González and Veas, 2019), but also in the elderly (Roch et al., 2020; Andrés-Roqueta et al., 2021).

Some studies deal with paralinguistic recognition and production of emotions in relation to certain cognitive aspects in children and adolescents (Joyce et al., 2006; Amadó et al., 2016; Djordjevic et al., 2016a; Pochon et al., 2017; Barisnikov et al., 2020). However, we are not familiar with studies examining which of these aspects predict mentioned abilities in people with ID. The authors of previous research studies associate the obtained results, which show difficulties in understanding and producing emotions in people with moderate ID (MID^2^), with deficits in language and cognitive abilities (Djordjevic et al., 2016a). However, we did not find any manuscripts that examined the predictors of these abilities in adults with MID.

Based on this literature survey, we consider to be interesting to examine the relation between these skills and acoustic features of voice, intellectual ability, and receptive language skills in adults with ID. The topicality of this subject is also reflected in our everyday reality, where we face the global effects of the pandemic on the clinical population due to social distancing and limited social contacts. Thus, the practical value of this manuscript is even greater in this vulnerable population.

This research aimed to determine how different variables related to understanding and producing emotions predicted these skills in adults with MID.

The aim of this manuscript was to determine which of the mentioned variables – receptive language skills, voice acoustics, intelligence, and social-demographic variables (gender, age, etiology, level of intellectual functioning) were the best predictors of paralinguistic comprehension and production of emotions in communication in adults with MID.

## Materials and Methods

### Sample

The sample included 41 adults with MID, 20–49 years of age (*M* = 34.34, SD = 7.809). There were 17 (41.5%) male and 24 (58.5%) female participants in the sample. With regard to etiology, the sample included participants with MID of unknown etiology (*N* = 29) and participants with DS (*N* = 12). These two subgroups did not significantly differ in gender [*t*(39) = 0.016, *p* > 0.05] and age [*t*(39) = −2.018, *p* > 0.05]. All participants are native speakers of Serbian. After the initial consent from parents, we obtained data on the participants’ level of intellectual functioning from their medical records. Medical records provided information about the level of intellectual disability (i.e., the information that the participant functioned at the MID level) for each participant included in the sample. Their medical records also showed that different psychiatrists used different assessment tests at different times and referred to different diagnostic classifications. Therefore, we did not use the IQ data for the purpose of this study, but only the level of ID.

### Procedure

Prior to conducting the research, we obtained the approval of the Ethics Committee of the Faculty of Special Education and Rehabilitation, University of Belgrade (no. 109/1). After obtaining the approval, a request for conducting the research was sent to managers of three day-care centers for adults with disabilities in Belgrade. The request included a detailed explanation of the aim, sample, instruments, and procedure of the research. After obtaining their consent, the request was forwarded to special educators employed in day-care centers, and they contacted the user’s parents.

Contacted participants’ parents or guardians voluntarily signed informed consent for the participants to take part in the research, after which persons with MID also gave their consent. Only those participants who gave their consent to participate were included in the research. The participants were explained that they could leave the research at any time. First, a triage assessment of the participants was conducted. On the basis of anamnestic data, the inclusion criteria were the following: diagnosed moderate ID, absence of autism spectrum disorder characteristics, absence of associated psychiatric disorders and/or illnesses which could affect voice characteristics, age between 18 and 55 (absence of aging voice influence).

The research was conducted in day-care centers^3^ for adults with disabilities in Belgrade, whose services the participants used. The assessment was conducted in a speech therapy room, isolated from noise and distractors.

The participants were first given an explanation about the nature of the task. The Peabody scale and Raven scale were presented to participants using pictures.

The Paralinguistic scale was presented to participants on a computer. On the basis of the presented videos and questions asked questions, the participants answered the questions, and the examiner recorded their answers on a special answer sheet.

Phonation of the vowel/a/for the acoustic analysis was recorded directly on the computer through a microphone placed at a specific distance from the participant’s mouth.

### Research Instruments

#### Assssment of Paralinguistic Recognition of Emotions

The Paralinguistic scale from The Assessment Battery for Communication (Sacco et al., 2008), which consists of Paralinguistic comprehension of emotions and Paralinguistic production of emotions, was used to assess paralinguistic ability to recognize emotions. The complete battery was translated from Italian into Serbian with a double-blind method, so that the professor translated the Italian into Serbian, and again the court interpreter translated the Serbian translation back into Italian. Then both versions were compared and finally corrected. Of the total translated batteries, we used the translated two subscales.

Paralinguistic comprehension of emotions includes videos in which actors express emotions by speaking in a fictional language, and participants have to recognize those emotions (e.g., the actor makes angry hand gestures and grimaces while speaking in a fictional language, and the question is: “In your opinion, which emotion is the actor expressing, how does he/she feel?”). The participant then chooses one of five given answers (e.g., “happy, surprised, angry, sad, something else”). The videos were presented via laptop. The videos lasted between 20 and 25 s. It was possible to get a grade of 0 or 1 for each task, depending on whether the answer was correct (score = 1) or incorrect (score = 0). The respondent chooses one of the five offered answers. This subscale includes eight items and the total score is eight points. Paralinguistic production of emotions includes eight items in which participants should react to examiner’s request (e.g., “Ask me what time it is. Ask me as if you were upset.”). The items we used included requests involving different emotions (bored, upset, disturb, happy, angry, sad and scary). The same scoring principle applied to this subscale. The total score in this subscale is eight points (see Supplementary Material).

The total value of the Cronbach’s alpha coefficient for the Paralinguistic scale is 0.70 according to the authors of this scale (Bosco et al., 2012). The Paralinguistic scale has previously been used in the Serbian-speaking area in the population of people with ID, and it had a satisfactory value of the Cronbach’s alpha coefficient, which was 0.94 for the subscale of paralinguistic production, and 0.79 for the subscale of paralinguistic comprehension (Djordjevic et al., 2016b). In our research it was 0.617 for the subscale of paralinguistic comprehension, and 0.767 for the subscale of paralinguistic production.

#### Assessment of Receptive Language Skills

*Peabody Picture Vocabulary Test*, PPVT – 4 (Dunn and Dunn, 2007) – Peabody Picture Vocabulary Test was used to assess receptive language skills. This test includes 227 items, divided into 19 categories of 12 words. Testing is conducted by presenting the participants with four different pictures and instructing them to point to the picture which corresponds to the word said by the examiner. Two trials are given first, followed by testing in sets. Electronic version of the test was used in this research, and the pictures were shown on computer screen. Testing was stopped when a participant gave eight incorrect answers in one set. Raw score in this test was obtained by subtracting the total number of incorrect answers from the total number of items.

The Peabody test has high internal reliability, ranging from 0.92 to 0.98 (Dunn and Dunn, 2007). This instrument has previously been used in the Serbian-speaking area in the population of people with ID and it had satisfactory values (Djordjevic et al., 2018). In our research it was 0.995.

#### Assessment of Acoustic Voice Characteristics

Computerized Speech Lab (“*Kay Elemetrics” Corp.*, model 4300), Multi-Dimensional Voice Program (MDVP) – MDVP software was used for the analysis of acoustic voice characteristics. This program provides a detailed graphical and numerical presentation for 33 parameters. The examiner instructed the participants to sound the vowel *a* calmly and spontaneously, for 3–4 s. In accordance with the author’s recommendations, this procedure was repeated three times in order to select a voice of the best quality. Sony ECM-T150 microphone was placed at a distance of 5 cm from the participant’s mouth. The signal was recorded directly on the computer. In this research, the following acoustic parameters were analyzed: frequency variability parameters (F0, Jitt, and PPQ), amplitude variability parameters (Shim, vAm, and APQ), voice interruption parameter (DVB), noise and tremor estimation parameters (NHR, VTI, and SPI).

Multi-Dimensional Voice Program is often used for voice assessment in different population of participants in the Serbian-speaking area (Petrovic-Lazic et al., 2009, 2014).

Factor analysis, with Promax rotation, was used in this research to single out latent dimensions which summarize the parameters of acoustic voice characteristics (Table 1). The analysis of main components, with the principle of distinguishing only those dimensions with eigenvalues greater than 1, singled out three factors. Together, they explain 80% of the variance in individual differences with regard to voice.

**TABLE 1:** Matrix of the results of analyzing main components used for acoustic voice characteristics with Promax rotation.

	Factors			
	1	2	3	
Shim	**1.033**	−0.223		
APQ	**1.014**	−0.173		
Jitt	**0.811**		0.326	
PPQ	**0.796**	0.122	0.328	
VTI	**0.754**	0.110	−0.503	
NHR	**0.628**	0.429	−0.125	
F0	−0.296	**1.017**		
DVB	0.171	**0.598**		
SPI			**0.869**	
vAm	0.330		**0.649**	
% variance	50.103	17.224	12.010	79.337

*1 – voice perturbations, 2 – voice frequency and interruptions, 3 – noise and tremor in voice. Shim, amplitude variation of the sound wave; APQ, amplitude perturbation quotient; Jitt, frequency variation; PPQ, pitch period perturbation quotient; VTI, voice turbulence index; NHR, noise-to-harmonic ratio; F0, mean fundamental frequency; DVB, degree of voice break; SPI, soft phonation index; vAm, peak amplitude variation. Bold values are factor loadings >0.50.*

The obtained results are in accordance with the Scree plot chart, which also recommends that three dimensions be singled out. The first factor includes the following variables: Shim, APQ, Jitt, PPQ, VTI, and NHR (voice perturbations). This factor explains 50% of the variance. The second factor includes F0 and DVB variables (voice frequency and interruptions), and it explains additional 17% of the variance. The third factor includes SPI and vAm variables (noise and tremor in voice). It explains additional 12% of the variance. The obtained three factors (voice perturbations, voice frequency and interruptions, noise and tremor in voice) will be used in further analyses.

The Cronbach’s alpha coefficient for the acoustic parameters used in our research was 0.856.

#### Assessment of Intellectual Functioning

Raven’s Progressive Matrices (Raven and Raven, 1998) – Raven’s progressive matrices were used to assess intellectual functioning. This instrument consists of non-verbal tasks which measure the general intelligence factor. The tasks are organized in such a way that there is a pattern, but with one segment always missing. The participant has to discover a rule according to which the patterns are arranged, and choose the one that is missing form several given options. The matrix consists of 60 tasks arranged in five sets. Arrangement of tasks is based on difficulty. Sets of tasks are arranged with regard to topics (supplementing, finding analogy, changing, permutation, and division). Electronic version of the matrix was used for the purpose of this research. The participants were first explained that an element was missing at the top of the page, while the answers were given at the bottom of the page. After selecting an answer at the bottom, participants pointed with their finger and the number of the answer was recorded. Each participant first did a trial, after which the assessment began. All correct answers were added up to get a total raw score, which shows the level of intellectual functioning.

The Cronbach’s alpha coefficient for this scale in our research was 0.815.

## Results

### Participants’ Results on All Applied Scales

Table 2 shows descriptive statistics of participants’ achievements in all analyzed variables. The rows show the variables, and the columns show the determined data values – the number of the participants who answered the questions, the score range of the variables, mean, standard deviation, skewness and its standard error, kurtosis and its standard error. Raven’s progressive matrices scores were within the expected range given the examined population. High deviation of skewness and kurtosis parameters and their standard errors, which indicates not meeting the conditions for normal distribution of results, was in our manuscript observed in certain variables from the Acoustic voice characteristics domain: Shim, DVB, NHR, and PPQ (Table 2).

**TABLE 2:** Descriptive measures of all variables used in the manuscript.

	*N*	Min	Max	*M*	SD	Sk		Ku	
(1)	41	20	49	34.34	7.809	0.195	0.369	−0.636	0.724
(2)	41	32	172	106.15	36.241	−0.191	0.369	−0.511	0.724
(3)	41	8	24	13.78	3.560	0.815	0.369	0.744	0.724
(4)	41	0	8	2.61	2.312	0.580	0.369	−0.585	0.724
(5)	41	3	15	9.37	2.791	−0.151	0.369	−0.005	0.724
(6)	41	3	19	11.98	4.083	−0.313	0.369	−0.737	0.724
**Acoustic features of voice**									
(7)	41	3.42	22.48	11.12	4.592	0.514	0.374	−0.116	0.733
(8)	41	6.60	82.58	23.83	14.203	2.068	0.374	6.657	0.733
(9)	41	2.84	15.98	8.25	3.416	0.623	0.374	−0.169	0.733
(10)	41	0.172	13.52	4.13	3.311	0.875	0.374	0.091	0.733
(11)	41	0.10	8.31	2.66	2.208	0.880	0.374	−0.218	0.733
(12)	41	0.00	52.79	2.83	9.689	4.320	0.374	19.848	0.733
(13)	41	0.17	1.67	0.39	0.265	3.005	0.374	13.597	0.733
(14)	41	0.039	1.29	0.27	0.260	2.673	0.374	7.802	0.733
(15)	41	0.26	4.642	1.57	0.967	1.241	0.374	1.908	0.733
(16)	41	14	77	41.83	14.874	0.061	0.369	−0.183	0.724

*N - number of participants; Min - participant’s lowest achievement; Max - participant’s highest achievement; M - arithmetic mean; SD - standard deviation; Sk - Skewness; Ku - Kurtosis. (1) Age; (2) Peabody; (3) Raven score; (4) Paralinguistic production of emotion; (5) Paralinguistic comprehension of emotion; (6) Total Paralinguistic score; (7) F0; (8) Shim; (9) vAm; (10) APQ; (11) Jitt; (12) PPQ; (13) DVB; (14) NHR; (15) VTI; (16) SPI.*

Table 3 shows the correlation coefficients of all used variables. The rows show numbered variables, and their names are shown in the description of columns. Spearman’s correlation coefficients are shown in the matrix cells. The variables of the same domain highly correlate with each other.

**TABLE 3:** Intercorrelation of all used variables.

		(1)	(2)	(3)	(4)	(5)	(6)	(7)	(8)	(9)	(10)	(11)	(12)	(13)	(14)	(15)
(1)	−1															
(2)	−2	–0.11														
(3)	−3	–0.01	0.464**													
(4)	−4	0.01	0.506**	0.339*												
(5)	−5	–0.04	0.457**	0.24	0.28											
(6)	−6	–0.02	0.599**	0.354*	0.754**	0.839**										
(7)	−7	0.26	–0.15	–0.24	0.09	–0.06	0.01									
(8)	−8	0.13	0.02	–0.2	–0.04	0.17	0.09	–0.02								
(9)	−9	0.1	–0.28	–0.21	–0.23	−0.358*	−0.366*	0.11	0.331*							
(10)	−10	0.16	0.04	–0.16	0.01	0.16	0.11	0.02	0.981**	0.370*						
(11)	−11	0.13	–0.07	−0.327*	–0.13	–0.07	–0.12	0.21	0.772**	0.583**	0.796**					
(12)	−12	0.19	–0.06	−0.323*	–0.1	–0.04	–0.09	0.23	0.792**	0.556**	0.826**	0.982**				
(13)	−13	0.2	0.03	–0.29	0.16	0.11	0.17	0.374*	0.340*	0.03	0.377*	0.27	0.395*			
(14)	−14	–0.03	–0.07	–0.25	0.01	–0.02	–0.01	0.462**	0.607**	0.29	0.603**	0.619**	0.624**	0.417**		
(15)	−15	–0.11	0.02	–0.1	0.12	0.14	0.16	0.22	0.590**	0.13	0.580**	0.459**	0.433**	0.24	0.775**	
(16)	−16	–0.15	0.1	0.06	0.04	–0.03	0	–0.09	0.03	0.356*	0.05	0.2	0.21	–0.07	–0.06	−0.358*

**p < 0.01, *p < 0.05. (1) Age; (2) Peabody; (3) Raven score; (4) Paralinguistic production of emotion; (5) Paralinguistic comprehension of emotion; (6) Total Paralinguistic score; (7) F0; (8) Shim; (9) vAm; (10) APQ; (11) Jitt; (12) PPQ; (13) DVB; (14) NHR; (15) VTI; (16) SPI.

The table shows that acoustic voice characteristics highly correlate with each other. Paralinguistic production of emotions and paralinguistic comprehension are highly interrelated. Also, Raven and Peabody are highly interrelated.

### Predictors of Paralinguistic Comprehension of Emotions

Hierarchical regression analysis was used to determine the predictors of paralinguistic comprehension of emotions (Table 4). This analysis evaluated the possibility to predict paralinguistic comprehension of emotions by the set of variables used in the research (gender, age, etiology, level of intellectual functioning, receptive language skills), as well as by three factors of acoustic voice characteristics (voice perturbation, voice frequency and interruptions, noise, and tremor in voice). The predictors were introduced hierarchically in blocks. The first block included gender, age, and etiology. The second block included variables from the domain of receptive language skills, level of intellectual functioning, and which were added to the variables from the first block. The third block included variables from the domain of acoustic voice characteristics which were grouped according to the results of factor analysis (Table 1).

**TABLE 4:** Results of hierarchical regression analysis for predicting paralinguistic comprehension of emotions.

Block		B	*t*	*p*	*R*	*R* ^2^	*F*(3/37)	*P*
Block I	Gender	–0.037	–0.219	0.828	0.208	0.043	0.558	0.646
	Age	–0.169	–1.000	0.324				
	Etiology	0.108	0.668	0.508				
Block II	Gender	0.015	0.089	0.929	0.420	0.176	1.213	0.323
	Age	–0.082	–0.467	0.644				
	Etiology	0.145	0.733	0.468				
	Raven score	–0.100	–0.477	0.636				
	Peabody	0.468	2.236	**0.032**				
Block III	Gender	0.008	0.046	0.963				
	Age	–0.109	–0.629	0.534				
	Etiology	0.093	0.465	0.645				
	Raven score	0.058	0.260	0.796	0.524	0.275	1.306	0.274
	Peabody	0.436	2.110	**0.043**				
	Voice perturbations	0.217	1.212	0.235				
	Voice frequency and interruptions	–0.064	–0.380	0.707				
	Noise and tremor in voice	0.201	1.151	0.259				

*β – standardized regression coefficient, t – significance parameter of standardized regression coefficient, p – statistical significance of standardized regression coefficient, R – regression model for predicting paralinguistic comprehension of emotions with hierarchical introduction of gender, age, and etiology (first level), Raven and Peabody (second level) and factor scores of acoustic voice characteristics (third level), R^2^ – variance percentage of paralinguistic comprehension of emotions explained with hierarchical regression model, F – parameter of statistical significance of regression model, P – statistical significance of regression model. Bold values p < 0.05.*

The results of this regression analysis singled out two significant models. The first explains about 4% of the criteria variance [R^2^ = 0.043, F(3/37) = 0.558, *P* > 0.05], and the second explains about 17% [R^2^ = 0.176, F(3/37) = 1.213, *P* > 0.05]. In this analysis, receptive language skills proved to be a significant predictor of paralinguistic comprehension of emotions (β = 0.468, *t* = 2.236, *p* < 0.05).

### Predictors of Paralinguistic Production of Emotions

Hierarchical regression analysis was applied to determine the predictors of paralinguistic production of emotions (Table 5). This analysis evaluated the possibility to predict paralinguistic production of emotions by the set of variables used in the research (gender, age, etiology, level of intellectual functioning, receptive language skills), as well as by three factors of acoustic voice characteristics (voice perturbation, voice frequency and interruptions, noise and tremor in voice). The predictors were introduced hierarchically in blocks, in the same way as in the process of determining the predictors of paralinguistic comprehension of emotions.

**TABLE 5:** Results of hierarchical regression analysis for predicting paralinguistic production of emotions.

Block		β	*t*	*p*	*R*	*R* ^2^	F (3/37)	*P*
Block I	Gender	0.089	0.535	0.596	0.270	0.073	0.972	0.416
	Age	–0.173	–1.043	0.304				
	Etiology	0.228	1.433	0.160				
Block II	Gender	0.067	0.476	0.637	0.639	0.409	3.918	**0.004**
	Age	–0.092	–0.621	0.539				
	Etiology	–0.072	–0.431	0.670				
	Raven score	0.213	1.200	0.238				
	Peabody	0.303	1.709	0.097				
Block III	Gender	–0.006	–0.041	0.968	0.691	0.477	4.301	**0.002**
	Age	–0.106	–0.743	0.463				
	Etiology	–0.057	–0.357	0.724				
	Raven score	0.270	1.578	0.124				
	Peabody	0.298	1.763	0.087				
	Voice frequency and interruptions	0.280	2.076	**0.046**				

*β – standardized regression coefficient; t – significance parameter of standardized regression coefficient; p – statistical significance of standardized regression coefficient; R – regression model for predicting paralinguistic production of emotions with hierarchical introduction of gender, age, and etiology (first level), Raven and Peabody (second level) and factor scores of acoustic voice characteristics (third level); R^2^ – variance percentage of paralinguistic production of emotions explained with hierarchical regression model; F – parameter of statistical significance of regression model; P – statistical significance of regression model. bold values p < 0.05, bold values P < 0.05.*

Regression analysis singled out three significant models. The first explains about 7% [R^2^ = 0.073, F(3/37) = 0.972, *P* > 0.05], the second about 40% [R^2^ = 0.409, F(3/37) = 3.918, *P* < 0.01], and the third 48% [R^2^ = 0.477, F(3/37) = 4.301, *P* < 0.01] of criteria variance. In this analysis, the second factor from the domain of acoustic voice characteristics (voice frequency and interruptions) proved to be a significant predictor of paralinguistic production of emotions. (β = 0.280, *t* = 2.076, *p* < 0.05).

## Discussion

Earlier manuscripts on paralinguistic comprehension and production of emotions in people with ID assumed that these abilities were related to language and cognitive deficits. However, we are not familiar with manuscripts that more precisely determine which language and cognitive domains best predict these abilities. Our manuscript examined whether specific acoustic voice characteristics, receptive language skills, gender, age, etiology, and the level of intellectual functioning had a predictive value in paralinguistic comprehension and production of emotions in adults with MID.

Regression analysis showed that, from three hierarchical groups of predictors, only receptive language skills from the second group were a significant predictor of paralinguistic comprehension of emotions. Interestingly, receptive language skills were a significant predictor of understanding emotions, although the actors in our study spoke a non-existent language while expressing basic emotions. In addition to the required answers, the researchers also noted down the comments of the participants (which were not included in this analysis) who tried to detect the language the actor spoke (e.g., “I know, he is speaking Japanese”) or tried to “translate” the spoken message (e.g., “He said that he was sad and that he was crying because of that”). All this indicates that people with MID in our sample largely relied on the meaning of the spoken messages, even when they did not mean anything. Receptive language skills measured by the Peabody test for assessing receptive speech are not limited by expressive language abilities or working memory limitations (Loveall et al., 2016). Thus, we can assume that receptive language skills measured by the Peabody receptive speech test are a measure of lower, perceptual cognitive abilities compared to the level of intellectual functioning measured by Raven’s progressive matrices that measures higher, more complex forms of cognition. Pochon et al. (2017) showed that the measures of intellectual functioning obtained by Raven’s progressive matrices did not predict the ability to understand emotions in adolescents with DS, unlike in typically developing adolescents. According to Beck et al. (2012), receptive language skills refer to coded verbal concepts gained from experience in relation to the environment. The same authors state the possibility that recognizing emotions is closely related to receptive language skills since the conceptualization of emotions may arise from lexical-semantic differentiation. Another possible explanation is that there is a common conceptualization mechanism that connects receptive language skills and emotion recognition (Beck et al., 2012). The fact that these abilities are conditioned by learning and experience can also be the reason why we did not get significant results for Raven’s matrices measures. They are non-verbal cognitive measures that refer to general intelligence (Raven and Raven, 1998) unrelated to experience and learning (Cattell, 1971). Furthermore, Joyce et al. (2006) showed that adults with ID were better in recognizing emotions than in producing them, and that these abilities were related to receptive language skills. These findings confirmed previous ones on the importance of receptive language skills (Dagnan et al., 2000).

With regard to paralinguistic production, the participants in our research were required to utter specific content and show a specific emotion in the way they speak (e.g., “Tell me to close the door. Say it angrily.”; “Ask me where the doctor is. Do it sadly”). The results of regression analysis in predicting paralinguistic production of emotions showed that the second factor in the acoustic voice characteristics domain (frequency and interruptions) from the third group was a significant predictor of paralinguistic production of emotions. This factor refers to the mean value of fundamental frequency and the percentage of parts with voice interruptions. In one research (Mendhakar et al., 2019), these parameters proved to be significantly different in premature babies compared to term babies. DVB represents the relation between the total duration of the parts with voice interruptions and the duration of the complete voice sample. It was shown that high-risk babies had a higher degree of voice interruptions. A higher degree of interruptions indicates bigger pauses and distribution of non-vocal components in a child’s cry (Mendhakar et al., 2019). The authors assume that these characteristics are attributed to immature larynx innervation in premature babies (Mendhakar et al., 2019). Research into acoustic voice characteristics in adults with ID of unknown etiology and DS shows that these people have a smaller range of frequency variability, which indicates voice monotony (Gautam and Singh, 2016; Corrales-Astorgano et al., 2018). As an element of prosody, F0 has a significant paralinguistic function (Ní Chasaide and Gobl, 2004). Difficulties in producing emotions can be associated with monotonous and less melodic speech in people with ID. Certain intonation-melodic variability and a larger range of fundamental voice frequency are necessary to express emotions. It is assumed that structural differences in the larynx level become more pronounced in adulthood (O’ Leary et al., 2019) and can be related to emotion production.

Emotion production is a more complex ability than emotion comprehension and, apart from perception, requires the use of non-linguistic (prosodic) elements of speech (Scherer, 2003). The assessed receptive language skills and the level of intellectual functioning were measured by scales that require answering based on recognition only, without expressive production. Therefore, it was expected that they did not prove to be significant predictors. This was also assumed by the author whose research showed no significant correlation between paralinguistic production of emotions and the level of intellectual functioning measured by Raven’s progressive matrices (Djordjevic et al., 2016a).

According to Beck et al. (2012), people with more developed lexical abilities are more successful in the general conceptualization of verbal concepts, and thus probably in the conceptualization of emotions. Many research studies show that people with DS have significantly more pronounced deficits in the linguistic domain compared to their cognitive capacities (Laws and Bishop, 2004; Zampini et al., 2016). Therefore, the sample size in this manuscript may be the reason why etiology did not have a significant influence as a variable that significantly predicted the criteria variables. This was also confirmed by other studies such as Wishart et al. (2007), who compared the ability to recognize emotions in participants with ID of different etiology and found that only children with DS had worse results than typically developing children. A significant positive correlation was also found between receptive language skills measured by the Peabody picture test and recognizing certain emotions from facial expressions in adults with Down syndrome (DS) as opposed to the control group (typically developing adults) (Hippolyte et al., 2008). The authors assumed that some emotional expressions required more complex semantic representation but preferred to explain that by a specific emotional deficit characteristic of participants with DS.

An older study by Leung and Singh (1998) showed that adults with ID had a poorer ability to recognize emotions than typically developing children. Also, in a sample of adults with mild and moderate ID, it was shown that younger participants and people with milder ID recognized emotions better (McKenzie et al., 2000). This was also confirmed by Matheson and Jahoda (2005), who found that the ability to recognize emotions decreased with age in adults with ID. Possibly a more even age structure of the participants and a bigger sample would influence the obtained results, which could be a recommendation for future research.

## Limitations

This research has several limitations. One refers to sample size, and future studies should include many more participants so that the results could be generalized. Apart from the size, another sample-related limitation is the participants’ structure. Thus, future studies should include more participants with known etiology, classified into groups with different syndromes. The application of tests that require different levels of cognitive information processing could be expanded in future research by using additional tests at the same level of processing. In addition to verbal tasks, accompanying pictographic material should also be included in assessing people with ID for the purpose of understanding orders.

Only one instrument was used in this research to assess comprehension and production of emotions. Thus, conclusions should be drawn with caution and future studies should verify these results using additional instruments.

## Conclusion

The results obtained in our study showed that receptive language skills had a predictive value in paralinguistic comprehension of emotions, and voice frequency and interruptions, from the domain of acoustic voice characteristics, predicted paralinguistic production of emotions.

Since recognizing emotions is the basis of social interaction, as pointed out by many authors, it is important to encourage the skills which are most closely related to comprehension and production of emotions so that they could develop as well. Much more research in this area is needed before more precise educational and therapeutic guidelines can be established. With regard to this, future studies could go in the direction of longitudinal monitoring of these abilities in people with MID. Also, more precise measures of the level of intellectual functioning, in addition to Raven’s Progressive Matrices, should be included in further analyses.

## Data Availability Statement

The original contributions presented in this study are included in the article/Supplementary Material, further inquiries can be directed to the corresponding author.

## Ethics Statement

The studies involving human participants were reviewed and approved by Ethics Committee of the Faculty of Special Education and Rehabilitation, University of Belgrade (no. 109/1). The patients/participants provided their written informed consent to participate in this study.

## Author Contributions

All authors listed have made a substantial, direct, and intellectual contribution to the work, and approved it for publication.

## Conflict of Interest

The authors declare that the research was conducted in the absence of any commercial or financial relationships that could be construed as a potential conflict of interest.

## Publisher’s Note

All claims expressed in this article are solely those of the authors and do not necessarily represent those of their affiliated organizations, or those of the publisher, the editors and the reviewers. Any product that may be evaluated in this article, or claim that may be made by its manufacturer, is not guaranteed or endorsed by the publisher.

## Footnotes

1 Down syndrome (DS) is the most common genetic cause of intellectual disability. The overall prevalence varies from 1 in 800 to 1,200. Most people with DS function within the mild to moderate ID range. These people have underdeveloped language and communication skills primarily due to speech delays, poor articulation due to narrow oral cavity, and not understanding language (Agarwal Gupta and Kabra, 2013).

2 There are four basic ID levels: mild, moderate (MID), severe, and profound. About 10% of people with ID function at the MID level. According to the DSM-4, persons with moderate ID have IQ in the range of 35–49 IQs. Description of this condition indicates that these people can take care of themselves, travel to familiar places in their community, and learn basic skills related to safety and health. Their self-care requires moderate support. According to all these previous statements, it can be concluded that MID can perform most daily activities independently with occasional support (American Psychiatric Association, 2013). Although they can be successful in basic communication, they can have difficulties understanding and comprehending non-verbal and subtle signals in social situations.

3 Day-care services in Serbia are social protection services for children and young people with physical impairment and intellectual disability. According to the current Policy, the purpose of day-care services is to improve the quality of life of users in their social environment through maintaining and developing social, psychological, and physical functions and skills, in order to enable them to live as independently as possible (The Policy was published in the “Official Gazette of RS, No. 42/2013 from 14th May 2013, and became effective on 22nd May 2013).
