# Supplementary material for: Searching for Best Predictors of Paralinguistic Comprehension and Production of Emotions in Communication in Adults With Moderate Intellectual Disability
Source: Front Psychol. 2022 Jul 8;13:884242. doi: 10.3389/fpsyg.2022.884242 (PMC9308010; doi:10.3389/fpsyg.2022.884242)
Supplement: Supplementary file 1 [file Data_Sheet_1.PDF]

Supplementary material

Paralinguistic comprehension			
Item		Scores	
Basic emotion	P1		
Expressive form	The participant understood the feeling presented by the character	0	1
Basic emotion	P4		
Expressive form	The participant understood the feeling presented by the character	0	1
Basic emotion	P3		
Expressive form	The participant understood the feeling presented by the character	0	1
Basic emotion	P8		
Expressive form	The participant understood the feeling presented by the character	0	1
Basic emotion	P5		
Expressive form	The participant understood the feeling presented by the character	0	1
Basic emotion	P6		
Expressive form	The participant understood the feeling presented by the character	0	1
Basic emotion	P7		
Expressive form	The participant understood the feeling presented by the character	0	1
Basic emotion	P2		
Expressive form	The participant understood the feeling presented by the character	0	1

Paralinguistic production			
Basic emotion	P21		
Expressive form	Participants responds with appropriate intonation/facial expression for the required feeling	0	1
Basic emotion	P22		
Expressive form	Participants responds with appropriate intonation/facial expression for the required feeling	0	1
Basic emotion	P23		
Expressive form	Participants responds with appropriate intonation/facial expression for the required feeling	0	1
Basic emotion	P24		
Expressive form	Participants responds with appropriate intonation/facial expression for the required feeling	0	1

Basic emotion	P 25		
Expressive form	Participants responds with appropriate intonation/facial expression for the required feeling	0	1
Basic emotion	P 26		
Expressive form	Participants responds with appropriate intonation/facial expression for the required feeling	0	1
Basic emotion	P 27		
Expressive form	Participants responds with appropriate intonation/facial expression for the required feeling	0	1
Basic emotion	P 28		
Expressive form	Participants responds with appropriate intonation/facial expression for the required feeling	0	1
